# Ni_3_S_2_–Ni Hybrid Nanospheres with Intra‐Core Void Structure Encapsulated in N‐Doped Carbon Shells for Efficient and Stable K‐ion Storage

**DOI:** 10.1002/advs.202205556

**Published:** 2023-01-01

**Authors:** Xiangtao Yu, Xiangyu Ren, Zhangfu Yuan, Xinmei Hou, Tao Yang, Mingyong Wang

**Affiliations:** ^1^ Collaborative Innovation Center of Steel Technology University of Science and Technology Beijing Beijing 100083 P. R. China; ^2^ State Key Laboratory of Advanced Metallurgy University of Science and Technology Beijing Beijing 100083 P. R. China

**Keywords:** electrodepositing nanosphere particle, intra‐core void, N‐doped carbon encapsulated, Ni_3_S_2_‐Ni hybrid, potassium ion battery

## Abstract

Iron group metals chalcogenides, especially NiS, are promising candidates for K‐ion battery anodes due to their high theoretical specific capacity and abundant reserves. However, the practical application of NiS‐based anodes is hindered by slow electrochemical kinetics and unstable structure. Herein, a novel structure of Ni_3_S_2_–Ni hybrid nanosphere with intra‐core voids encapsulated by N‐doped carbon shells (Ni_3_S_2_‐Ni@NC‐AE) is constructed, based on the first electrodeposited NiS nanosphere particles, dopamine coating outer layer, oxygen‐free annealing treatment to form Ni_3_S_2_‐Ni core and N‐doped carbon shell, and selective etching of the Ni phase to form intra‐core void. The electron/K^+^ transport and K^+^ storage reaction kinetics are enhanced due to shortened diffusion pathways, increased active sites, generation of built‐in electric field, high K^+^ adsorption energies, and large electronic density of states at Fermi energy level, resulting from the multi‐structures synergistic effect of Ni_3_S_2_‐Ni@NC‐AE. Simultaneously, the volume expansion is alleviated due to the sufficient buffer space and strong chemical bonding provided by intra‐core void and yolk–shell structure. Consequently, the Ni_3_S_2_‐Ni@NC‐AE exhibits excellent specific capacity (438 mAh g^−1^ at 0.1 A g^−1^ up to 150 cycles), outstanding rate performances, and ultra‐stable long‐cycle performance (176.4 mAh g^−1^ at 1 A g^−1^ up to 5000 cycles) for K‐ion storage.

## Graphical abstract

Novel structure of Ni_3_S_2_–Ni hybrid nanosphere with intra‐core voids encapsulated by N‐doped carbon shells (Ni_3_S_2_–Ni@NC‐AE) is constructed, based on first electrodeposited NiS nanosphere particles, dopamine coating outer layer, oxygen‐free annealing treatment to form Ni_3_S_2_–Ni core and N‐doped carbon shell, and selective etching of Ni‐phase to form intra‐core void. The Ni_3_S_2_‐Ni@NC‐AE exhibits excellent K‐ion storage performance due to the multi‐structure synergistic effect.

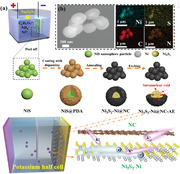

## 1 Introduction

Potassium‐ion batteries (PIBs) are considered as favorable replacement or supplementary candidates for lithium‐ion batteries in large‐scale energy storage systems due to their advantages, such as high abundance (K: 1.5 wt.% vs Li: 0.0017 wt.%), high cell voltage (K: −2.93 V vs standard hydrogen electrode (SHE) and Li: −3.04 V vs SHE), and high energy density due to weak Lewis acidity.^[^ ^1^, ^2^, ^3^ ^]^ However, the practical application of PIBs is hindered by the difficulty in developing suitable anode materials that can reversibly accommodate large size K‐ions.^[^ ^4^, ^5^ ^]^ Recently, huge progress has been made in the research of metals chalcogenides as anode materials for PIBs.^[^ ^6^, ^7^, ^8^ ^]^ Among them, Ni_y_S_x_ is considered as one of the greatest potential PIBs anode because of the preferable electrical conductivity due to weak Ni–S bond energy, high theoretical capacity induced by multiple electron reactions, and fast ion transport kinetics due to layered structure of Ni_y_S_x_.^[^ ^9^, ^10^ ^]^ Nevertheless, because of the large radius and heavy mass of K^+^, Ni_y_S_x_ anode materials still suffer from low electron/ion transport rates and reaction kinetics, as well as large volume expansion, which leads to poor rate performance and rapid capacity decay. It is a great challenge to develop Ni_y_S_x_ as reliable PIBs anode.

Nanosphere materials could reduce the electron/ion migration path^[^ ^5^, ^11^, ^12^ ^]^ and increase the reactive specific surface area for K^+^ storage^[^ ^13^ ^]^ due to quantum size and surface effect induced by ultra‐fine particles, thus exhibiting excellent performance.^[^ ^12^ ^]^ Electrodeposition is considered to be a promising technique for preparing nanosphere metal‐based materials due to its high efficiency, simplicity, and ability to mass‐produce products.^[^ ^14^, ^15^ ^]^ However, due to the high exchange current density of iron group metals leading to small electrocrystallization nucleation exclusion zone, large‐sized cauliflower‐like microspheres are usually obtained instead of nanosphere particles.^[^ ^16^, ^17^ ^]^ It is well known that electrodeposition particle size can be changed by additives which affect the nucleation exclusion zone.^[^ ^5^, ^18^ ^]^ Therefore, the selection of suitable sulfur source additives is the key to electrodeposit NiS nanospheres. Additionally, nanosphere structure increases interface electron/ion transfer resistance, and accentuates self‐aggregation and pulverization of particles during cycling.^[^ ^5^ ^]^ As we all know, the core‐shell structure encapsulating NiS‐based active species by N‐doped carbon layers can enhance electrical conductivity, accelerate K^+^ transport, boost K adsorption energy, and prevent particle agglomeration and pulverization to stabilize the structure due to its core–shell heterostructure interaction, such as formation of internal electric field and Ni–N bonds, etc.^[^ ^4^, ^7^, ^19^ ^]^ Furthermore, the intra‐core void structure can serve as a potassium reservoir for potassium storage to shorten the K^+^ transport path, increase the active area, provide a buffer space for volume expansion, and further improve the potassium storage activity and stability of the core–shell material.^[^ ^19^, ^20^ ^]^ Simultaneously, the synergistic effects of the Ni_3_S_2_–Ni hybrid structure can enrich the phase interface to accelerate the reaction kinetics and enhance the electrical conductivity.^[^ ^8^ ^]^

Herein, a facile and effective strategy for preparation of Ni_3_S_2_–Ni hybrid nanospheres with intra‐core void structure encapsulated in N‐doped carbon shells (Ni_3_S_2_–Ni@NC–AE) is developed, based on the electrodeposition of NiS nanospheres by regulating the electrocrystallization nucleation exclusion zone through Na_2_S_2_O_3_ acting as a sulfur source and dopamine coating. Then, Ni_3_S_2_–Ni dual‐phase hybrid structure and N‐doped carbon shell are formed by annealing, and intra‐core void structure is constructed by selective etching of Ni in the Ni_3_S_2_–Ni. The nanospheres structure can enhance the K storage electrochemical kinetics due to the quantum size and surface effect. Moreover, the Ni_3_S_2_–Ni hybrid structure can improve the electron/ion transfer because of the presence of abundant phase interfaces and metallic Ni phase. Additionally, the core–shell and intra‐core void structure can accelerate the reaction kinetics and enhance stability by improving electron/ion mass transfer and adsorption, and providing sufficient buffer space. As expected, the novel Ni_3_S_2_–Ni@NC‐AE PIBs anode manifests a high reversible specific capacity of 438.0 mAh g^−1^ at 0.1 A g^−1^ after 150 cycles, and superior long term cycling life of 176.4 mAh g^−1^ at 1 A g^−1^ after 5000 cycles.

## 2 Results and Discussion

**Figure 1:**
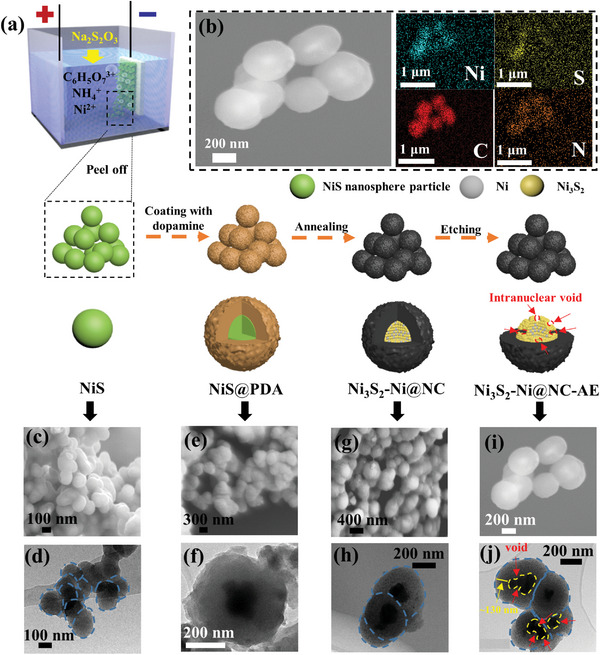
a) Schematic illustration of the preparation process for Ni_3_S_2_‐Ni@NC‐AE NPs. b) EDS elemental mapping of Ni_3_S_2_‐Ni@NC‐AE. c,e,g,i) SEM and d,f,h,j) TEM images of c,d) NiS, e,f) NiS@PDA, g,h) Ni_3_S_2_‐Ni@NC, and i,j) Ni_3_S_2_–Ni@NC‐AE.

The preparation process and formation mechanism of Ni_3_S_2_–Ni@NC‐AE NPs are shown in **Figure** **1**. First, a porous film composed of NiS NPs was electrodeposited for the first time by adding Na_2_S_2_O_3_ as sulfur source. Due to the poor binding force, dispersed NiS NPs can be obtained by simple mechanical peeling (Figure 1a,c,d; Figure S1, Supporting Information). Subsequently, the as‐prepared NiS NPs were coated with a layer of polydopamine by dopamine self‐polymerization (NiS@PDA).^[^ ^21^ ^]^ Then, the NiS@PDA was annealed at 450 °C under Ar/H_2_ atmosphere to construct Ni_3_S_2_–Ni hybrid core structure by phase separation (according to Ni–S phase diagram in Figure S1f, Supporting Information), and N‐doped carbon shell structure by polydopamine carbonization (Ni_3_S_2_‐Ni@NC). Finally, intra‐core void structure was created by selectively etching the Ni phase in Ni_3_S_2_–Ni in HCl solution, because the Ni_3_S_2_ phase with spinel structure possessed strong HCl etching resistance (Ni_3_S_2_–Ni@NC‐AE).^[^ ^22^, ^23^ ^]^

Generally, micron‐sized cauliflower‐like particles are easily obtained by electrodeposition of single‐metal Ni‐based alloys, because new nuclei are formed on immature nuclei. This is because the high melting point and low exchange current density of metal Ni lead to a small electrocrystallization nucleation exclusion zone.^[^ ^16^, ^24^ ^]^ However, when Na_2_S_2_O_3_ was introduced into the electrolyte, smooth NiS nanosphere particles with a diameter of 150–220 nm were electrodeposited for the first time (Figure 1c,d; Figure S1a–e, Supporting Information), indicating that the electrocrystallization nucleation exclusion zone increased.^[^ ^24^ ^]^ It may be due to the disproportionation reaction of Na_2_S_2_O_3_ during the electrodeposition process, which resulted in the non‐conductive S element adsorbing on the electrocrystallization nuclei surface and prevented the formation of new nuclei on immature nuclei. It is known that nanospheres can improve K storage kinetics by shortening the reaction path and increasing the specific surface area.^[^ ^5^ ^]^

As scanning electron microscopy (SEM) and transmission electron microscopy (TEM) images showed, the NiS@PDA surface became rough (Figure 1e), and the outside of NiS NPs was uniformly coated with a ≈130 nm‐thick polydopamine layer (Figure 1f) by polydopamine coating. After annealing, the nanosphere core‐shell structure was preserved (Figure 1g,h). There was no significant change in the size of Ni_3_S_2_‐Ni@NC (410–480 nm) compared with NiS@PDA. For Ni_3_S_2_‐Ni@NC‐AE, as energy dispersive X‐ray spectroscopy (EDS) mapping images showed (Figure 1i,b), Ni and S elements were mainly distributed in the central cores, and C and N elements were mainly distributed in the outer shells, confirming the NiS‐based cores were encapsulated in the N‐doped carbon shells. The size of Ni_3_S_2_‐Ni@NC‐AE remained unchanged obviously (410–480 nm) (Figure 1j). It was worth noting that the NiS‐based core size is basically unchanged for NiS@PDA and Ni_3_S_2_‐Ni@NC (150–220 nm), but decreased significantly for Ni_3_S_2_‐Ni@NC‐AE (60–140 nm). Additionally, many nanopores appeared in the NiS‐based core (arrows in Figure 1j), illustrating that intra‐core voids were formed after HCl solution etching. The well‐developed intra‐core void structure of Ni_3_S_2_‐Ni@CN‐AE could act as a K reservoir to shorten the K^+^ transport path, and buffer the drastic volume changes during the potassium/depotassium process, as well as increase the activity reaction area, thereby improving the specific capacity and stability for K storage.^[^ ^20^ ^]^

**Figure 2:**
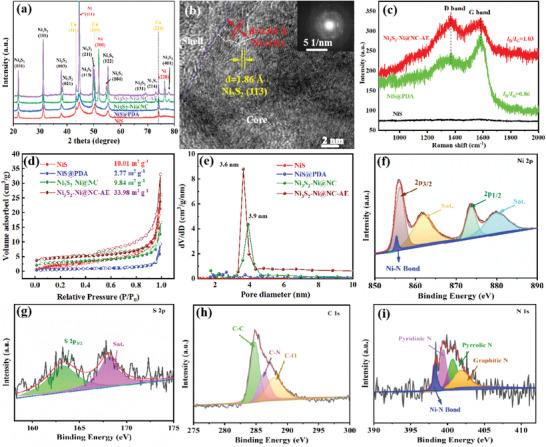
a) XRD diffractogram, b) HRTEM, c) Raman spectra, d) N_2_ adsorption–desorption isotherms curves, and e) the corresponding BJH pore‐size distribution plots of NiS‐based NPs. The high resolution XPS spectra of f) Ni 2p, g) S 2p, h) N 1s, and i) C 1s for Ni_3_S_2_‐Ni@NC‐AE.

The crystal structure evolution of NiS‐based NPs at different preparation steps was examined. For electrodeposited NiS, as X‐ray diffraction (XRD) results showed (**Figure** **2**a), weaker peaks appeared at 21.8°, 31.1°, 37.8°, 50.1°, 55.1°, and 44.5°, corresponding to the (101), (111), (003), (211), and (122) plane of Ni_3_S_2_ (JCPDS no. 44–1418)^[^ ^25^ ^]^ and (111) plane of Ni (JCPDS no. 87–0712),^[^ ^26^ ^]^ respectively. The weaker peak intensity indicated that the electrodeposited NiS was less crystalline. The HRTEM images showed no obvious lattice fringes (Figure S2a, Supporting Information), and selected electron diffraction diagram (SAED) pattern was a diffuse halo (Figure S2b, Supporting Information), which also verified that the electrodeposited NiS was mainly amorphous. The XRD pattern of NiS@PDA was basically consistent with that of electrodeposited NiS, indicating that the phase structure of NiS‐based NPs did not change after polydopamine coating. However, after annealing treatment (Ni_3_S_2_‐Ni@NC), the intensity of characteristic peaks was greatly enhanced. Moreover, in addition to the peaks appearing in NiS@PDA, six characteristic peaks (021), (113), (104), (131), (214), and (401) of Ni_3_S_2_ (JCPDS no. 44–1418) at 38.3°, 49.7°, 54.6°, 68.48°, 73.1°, and 77.9°,^[^ ^27^ ^]^ and two characteristic peaks (200), (220) at 51.9°, 76.4° of Ni (JCPDS no. 87–0712)^[^ ^28^ ^]^ appeared. Obvious lattice fringes with different distances ≈1.86 and 2.04 Å corresponding to (113) of Ni_3_S_2_ lattice and (111) of Ni lattice were found in the HRTEM of annealed NiS‐based NPs (Figure S3a, Supporting Information). Polycrystalline diffraction rings also appeared in the corresponding SAED pattern (Figure S3b, Supporting Information), which was consistent with the XRD result. The above results indicated that the annealed NiS‐based NPs had excellent crystallinity and the amorphous NiS phase separation into Ni_3_S_2_–Ni. Similar studies, such as the transformation of amorphous NiP into dual‐phase Ni_3_P‐Ni after annealing treatment have been reported.^[^ ^22^ ^]^ It could be predicted that the metallic Ni phase in Ni_3_S_2_–Ni hybrid structure could improve the electrical conductivity and rate performance, and could also be selectively etched to facilitate the formation of intra‐core void structures.^[^ ^22^, ^23^, ^29^ ^]^ The crystal structure of Ni_3_S_2_–Ni@NC‐AE was basically the same as unetched Ni_3_S_2_–Ni@NC (Figure 2b), except that the characteristic peak intensity corresponding to Ni was weakened (Figure 2a), which indicated that the Ni metal phase on the surface was selectively etched.^[^ ^22^, ^23^, ^29^ ^]^ Additionally, as the white dashed line in Figure 2b shows, obvious heterostructure interface between Ni_3_S_2_–Ni can be found, which was conducive to improving potassium storage kinetics.

The surface carbon information of NiS‐based NPs was characterized by Raman spectroscopy (Figure 2c). For electrodeposited NiS, there was no carbon band in the Raman spectrum, which was attributed to the absence of carbon in the outer layer of NPs. While, for NiS@PDA and Ni_3_S_2_‐Ni@NC‐AE, two peaks appeared at 1367.53 and 1583.9 cm^−1^ corresponding to the D (disordered carbon) and G (graphitic carbon) bands produced by the sp^3^ defective and sp^2^ ordered bond pairs, respectively. The D to the G band intensity ratios (*I*_D_/*I*_G_) of NiS@PDA and Ni_3_S_2_‐Ni@NC‐AE were 0.86 and 1.03, respectively. The result indicated that for Ni_3_S_2_‐Ni@NC‐AE, a large amount of disordered carbon with plentiful defects was formed on the outer layer, which was beneficial to enhance electron transfer.^[^ ^4^, ^25^ ^]^ The outer carbon shell content was determined according to the result of thermal gravimetric analysis (TGA), and the calculation details were provided in the Supporting Information. As Figure S4 (Supporting Information) shows, when Ni_3_S_2_‐Ni@NC‐AE was heated to 800 °C, the final product was NiO according to previous studies.^[^ ^30^ ^]^ The mass loss rate was ≈36.28 wt.%, and the carbon content could be estimated to be ≈35.77 wt.%. N_2_ adsorption–desorption tests were performed to probe the evolution of intra‐core void pore structure and specific surface area of NiS‐based NPs at different preparation steps. It could be found that N_2_ adsorption–desorption isotherms for NiS and NiS@PDA NPs were of type II (Figure 2d), indicating that the NPs are nonporous or macroporous structure. Combined with the absence of obvious peaks on the pore size distribution curves (Figure 2e), it could be further determined that the NiS and NiS@PDA NPs are non‐porous structure. It was noted that the adsorption–desorption isotherm curve of Ni_3_S_2_‐Ni@CN showed an obvious hysteresis loop, indicating that it was of type IV.^[^ ^31^ ^]^ The pore size distribution of Ni_3_S_2_–Ni@CN NPs was mainly located ≈3.9 nm, which illustrated that the NPs generated abundant mesoporous structure after annealing. For Ni_3_S_2_–Ni@CN‐AE, the highest *dV*/*dD* value appeared (Figure 2e), indicating that intra‐core void structure with an average pore size of ≈3.6 nm was constructed after etching, resulting in the largest nanopore volume. The Brunauer–Emmett–Teller (BET) specific surface area decreased from 10.01 m^2^ g^−1^ for electrodeposited NiS to 2.77 m^2^ g^−1^ for NiS@PDA, which was attributed to the increase of Ni‐based NPs size (from 150–220 to 410–480 nm) after polydopamine coating. After annealing, the specific surface area increased to 9.84 m^2^ g^−1^ (Ni_3_S_2_‐Ni@CN) due to the formation of mesoporous structure. For Ni_3_S_2_–Ni@CN‐AE, the specific surface area increased to 33.98 m^2^ g^−1^, further indicating the formation of inter‐core void structure after etching.

The chemical compositions and states of NiS‐based NPs surface were analyzed by X‐ray photoelectron spectroscopy (XPS). The survey XPS spectrum confirmed the presence of Ni, S, C, and N elements on the surface of NiS@PDA, Ni_3_S_2_–Ni@NC, and Ni_3_S_2_–Ni@NC‐AE NPs. While only Ni, S, and C elements were present on the surface of electrodeposited NiS NPs (Figure S5, Supporting Information), which was attributed to the presence of N‐containing functional groups in the PDA, thus an N‐doped carbon shell could be obtained after annealing.^[^ ^10^ ^]^ The S element content on Ni_3_S_2_‐Ni@NC‐AE surface increased from 0.16 to 0.32 at.% compared to Ni_3_S_2_–Ni@NC surface, which was consistent with the XRD results, again indicating that the metallic Ni was selectively etched. That is, the proportion of Ni_3_S_2_ phase with high theoretical capacity for K storage in Ni_3_S_2_–Ni core was increased. In high‐resolution Ni 2p spectrum (Figure 2f), two main peaks at ≈856.2 and 873.8 eV were assigned to Ni 2p_3/2_ and Ni 2p_1/2_, respectively. Two corresponding satellite peaks were located at 862.1 and 880 eV, respectively. This was consistent with the previously reported XPS spectra of Ni_3_S_2_–Ni dual‐phase coexistence.^[^ ^32^ ^]^ It was noting that the intensity of the metallic Ni (Ni^0^) peaks at 852.6  and 869.7 eV^[^ ^32^ ^]^ of Ni_3_S_2_–Ni@NC‐AE was significantly weakened compared to that of Ni_3_S_2_–Ni@NC, which further confirmed that Ni^0^ especially on the surface of Ni_3_S_2_–Ni core was selectively etched. In S 2p XPS spectrum (Figure 2g), two peaks at 163.3 and 168.0 eV corresponded to S 2p_3/2_ and S 2p_1/2_,^[^ ^33^ ^]^ respectively. The C 1s XPS spectrum could be decomposed into three peaks at 284.8, 286.3, and 288 eV, corresponding to C—C (sp^2^‐hybridized carbon), C—N, and C—O bonds, respectively (Figure 2h). The thermal decomposition of N‐containing and O‐containing groups in polydopamine formed C—N and C—O bonds, respectively.^[^ ^34^ ^]^ For N 1s spectrum (Figure 2i), peaks at 399.4, 400.7, and 401.8 eV, corresponded to pyridinic N, pyrrolic N, and graphitic N, respectively (Figure 2i). N mainly in the form of pyridine N meant that the lone pair N electrons contribute to the graphitic carbon, which enhanced the conductivity of the composite.^[^ ^35^ ^]^ Notably, Ni‐N bonds were observed in both Ni 2p (at 855.3 eV)^[^ ^36^ ^]^ and N 1s (at 398.3 eV)^[^ ^37^ ^]^ XPS spectra. Such a chemical bond existing between the NiS‐based active material and the N‐doped carbon shell could enhance structural stability during the potassium storage cycle and make the electron transport path more continuous.^[^ ^10^, ^38^ ^]^

**Figure 3:**
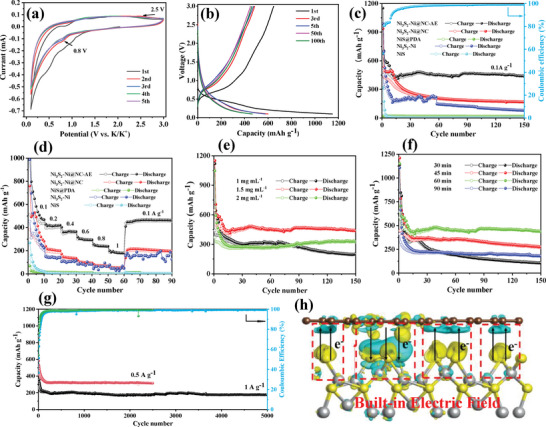
Potassium storage properties of NiS‐based NPs. a) CV curves of Ni_3_S_2_–Ni@NC‐AE for the first five turns at 0.2 mV s^−1^. b) Galvanostatic charge–discharge cycle curves of Ni_3_S_2_–Ni@NC‐AE for different cycles at 0.1 A g^−1^. c) Cycling performance of NiS‐based NPs for 150 cycles at 0.1 A g^−1^. d) Rate performance of NiS‐based NPs at varied current densities. Influence of e) dopamine concentration in coating solution and f) etching times on cycling performance of Ni_3_S_2_–Ni@NC‐AE. g) Long cycling performance of Ni_3_S_2_–Ni@NC‐AE at 0.5 and 1 A g^−1^. h) Charge difference density of Ni_3_S_2_@NC heterostructure (side view; electron annihilation: yellow; electron aggregation: green).

To evaluate the electrochemical performance of the NiS‐based NPs as anode for PIBs, coin‐type cells with potassium metal as both counter and reference electrodes were assembled. **Figure** **3**a showed the cyclic voltammetry (CV) curve of Ni_3_S_2_‐Ni@NC‐AE from 0.1 to 3 V at a 0.2 mV s^−1^. In initial cycle, a cathodic peak appeared at 0.6 V for the organic electrolyte decomposition and solid electrolyte interphase (SEI) film formation. The peak was significantly weakened in subsequent cycles, indicating that the electrolyte decomposition and SEI film formation mainly occurred in the first cycle.^[^ ^39^ ^]^ The cathodic peak at 0.8 V in subsequent cycles was caused by the reduction of Ni_3_S_2_ to Ni^0^,^[^ ^40^ ^]^ and the anodic peak at 2.5 V could be attributed to the oxidation of Ni^0^ and K_2_S to Ni_3_S_2_.^[^ ^41^ ^]^ The subsequent cycle curves had good overlap, indicating that Ni_3_S_2_‐Ni@NC‐AE possessed good reversibility during potassium/depotassium process. Moreover, it could be found that Ni_3_S_2_‐Ni@NC‐AE had a higher peak current and sharper peak compared to the electrodeposited NiS (Figure S9, Supporting Information), which illustrated that the Ni_3_S_2_‐Ni@NC‐AE had a good potassium storage reaction kinetics.^[^ ^42^ ^]^ As shown in Figure 3b, the initial discharge and charge capacities of Ni_3_S_2_‐Ni@NC‐AE at 0.1 A g^−1^ were 1150.3 and 647.7 mAh g^−1^, respectively, with a coulombic efficiency (CE) of 56.3%. The capacity loss in the first cycle could be attributed to the formation of SEI and the irreversible insertion of K^+^. The charge/discharge overlap curves of Ni_3_S_2_‐Ni@NC‐AE after the fifth cycle were significantly better than those of other NiS‐based NPs (Figure S10, Supporting Information), indicating the best reversibility. The excellent reversibility and stability of Ni_3_S_2_–Ni@NC‐AE was clearly benefited to the introduction of N‐doped carbon coating, inter‐core void, nanospheres, and Ni_3_S_2_–Ni hybrid structure. It was depicted that the intensity of redox peaks for CV curves was relatively weak (Figure 3a), and there were no evident voltage platforms in the charge‐discharge curves of NiS‐based electrode (Figure 3b), accounting for the large specific surface area and predominant transition metal compounds structure.^[^ ^1^, ^5^, ^7^, ^19^ ^]^ Such behaviors were consistent with the characteristics of transition metal sulfide (phosphides) KIBs anode materials.^[^ ^1^, ^5^, ^7^, ^19^ ^]^

In order to identify the enhanced effect of each structure of Ni_3_S_2_–Ni@NC‐AE on potassium storage performance, the cycling performance of NiS‐based NPs at different preparation stages was investigated at 0.1 A g^−1^ (Figure 3c). First, it was determined that the potassium storage reversible specific capacity of carbon paper substrate was only 0.05 mAh g^−1^ at 0.1 A g^−1^ (Figure S7, Supporting Information), illustrating that the specific capacity was mainly contributed by NiS‐based active materials. For electrodeposited NiS, although the first cycle specific capacity reached 317.8 mAh g^−1^, the specific capacity decayed rapidly thereafter, and it stabilized at ≈3.2 mAh g^−1^ after five cycles. After polydopamine coating (NiS@PDA), the cycling stability was improved, while the specific capacity was only ≈23.1 mAh g^−1^. For Ni_3_S_2_‐Ni hybrid NPs without any coating, the specific capacity decayed rapidly in the beginning few cycles, and then it fluctuated greatly in the following cycles, displaying poor cycling performance. It was worth noting that the specific capacity (≈207.2 mAh g^−1^ for 10 to 57 cycles and ≈109.5 mAh g^−1^ after 57 cycles) was significantly higher than that of NiS and NiS@PDA, which might attributed to the high conductivity and high theoretical potassium storage capacity of the Ni_3_S_2_–Ni hybrid structure. For Ni_3_S_2_‐Ni@NC, the specific capacity was ≈485.7 mAh g^−1^ for the first ten cycles, then decreased slowly and stabilized at ≈192.9 mAh g^−1^ after 60 cycles. The improved potassium storage performance could be attributed to the Ni—N bond between the N‐doped carbon shell and the NiS‐based active material, which enhanced the conductivity and stabilized the structure of the hybrid anode.^[^ ^10^ ^]^ Additionally, it could be found that in differential charge density maps calculated by density functional theory (DFT) (Figure 3h; Figure S12, Supporting Information), there was an inhomogeneous charge distribution between N‐doped carbon layer and Ni_3_S_2_ interface, which caused built‐in electric fields at the interface to accelerate charge separation and migration.^[^ ^43^, ^44^ ^]^ Thus, the diffusion kinetics of potassium storage was improved.^[^ ^45^ ^]^ After etching (Ni_3_S_2_–Ni@NC‐AE), the specific capacity first decreased rapidly to 447.2 mAh g^−1^, then increased slowly with the increase of cycle number, and stabilized at 467.7 mAh g^−1^ with a high CE of 99.2% after 90 cycles. The specific capacity increased slightly after cycling for a few turns in Figure 3e,f due to the activation of the porous electrode during the charge and discharge processes.^[^ ^46^, ^47^ ^]^ The further improvements of potassium storage performance (capacity and stability) benefited from the intra‐core void structure created after etching, which acted as a K reservoir to shorten the transmission path^[^ ^20^ ^]^ and a buffer to mitigate volume expansion.^[^ ^19^ ^]^ Moreover, as the SEM and TEM images of Ni_3_S_2_‐Ni@NC‐AE after 500 cycles at 0.1 A g^−1^ showed (Figure S13b–d, Supporting Information), the morphology of the anode was basically unchanged before and after the cycle, and the shell–core structure of the NiS‐based nanosphere particles was intact. The result further suggested that Ni_3_S_2_–Ni@NC‐AE had good structural stability.

As Figure 3d reveals, the specific capacities of NiS and NiS@PDA decayed rapidly with the increasing of current density, which were much lower than those of Ni_3_S_2_–Ni and Ni_3_S_2_–Ni@NC at varied current rates, indicating that Ni_3_S_2_–Ni hybrid structure and N‐doped carbon shell could significantly promote electron/ion transport and enhance reaction kinetics. Ni_3_S_2_–Ni@NC‐AE exhibited the best rate performance, which delivered high specific capacities of ≈482.1, 415.1, 369.3, 297.1, 240.9, and 182.7 mAh g^−1^ at 0.1, 0.2, 0.4, 0.6, 0.8, and 1A g^−1^, respectively, demonstrating that intra‐core void structure created by etching could further improve the potassium storage kinetics. When the current density returned to 0.1 A g^−1^, the specific capacity still reached 464.4 mAh g^−1^ with a capacity retention rate of 99%, showing good reversibility. Electrochemical impedance spectroscopy (EIS) analysis of the NiS‐based NPs was carried out to investigate the effects of the multi‐structure to the potassium storage performance (Figure S14, Supporting Information). There were two parts in all EIS Nyquist plots. A linear Warburg tail was in the low frequency region, and a semicircle was in the high‐to‐middle frequency domain. According to the semicircle diameter size in high frequency region, the interfacial resistance (*R*_ct_) was in the following order: Ni_3_S_2_–Ni@NC‐AE < Ni_3_S_2_–Ni@NC < NiS@PDA < NiS. Ni_3_S_2_–Ni@NC‐AE possessed the smallest reaction resistance, which might be attributed to the enhanced conductivity by the Ni_3_S_2_–Ni hybrid and intra‐core void structure. Meanwhile, Ni_3_S_2_–Ni@NC‐AE had the largest linear slope in low frequency region. The EIS results indicated that Ni_3_S_2_–Ni@NC‐AE possessed the best electrochemical kinetics compared to other samples.

From the above results, it could be found that N‐doped carbon shell and inter‐core void structure created by HCl etching could effectively improve the potassium storage performance of NiS‐based NPs. Herein, the effects of dopamine concentration in coating solution (i.e., N‐doped carbon shell thickness) and etching time (i.e., inter‐core void content) on the electrochemical properties were further investigated (Figure 3e,f). As Figure 1f and Figure S15 (Supporting Information) show, N‐doped carbon shell thickness increased from ≈10 to 170 nm with the dopamine concentration in coating solution increasing from 1 to 2 mg mL^−1^. For Ni_3_S_2_–Ni@NC‐AE with ≈10 nm N‐doped carbon shell, the specific capacity decayed rapidly after 60 stabilization cycles and dropped to 125.4 mAh g^−1^ after 150 cycles. It could be attributed to the poor encapsulation of the NiS‐based active material by the thinner carbon shell, which cannot effectively alleviate the huge volume expansion of the active material during long‐term cycling. Ni_3_S_2_–Ni@NC‐AE with 130 nm N‐doped carbon shell possessed the highest reversible capacity and better cycling performance (434.4 mAh g^−1^ after 150 cycles). However, further increasing the carbon shell thinness to 170 nm, the reversible capacity of Ni_3_S_2_–Ni@NC‐AE decreased to only 328.1 mAh g^−1^ after 150 cycles, although the cycle performance is very good. The results showed that the excessive carbon layer reduced the content of NiS‐based active material, which in turn reduced the specific capacity. As shown in Figure 1f, all Ni_3_S_2_–Ni@NC‐AE with different etching times had good cycling performance. As etching time increased from 30 to 60 min, the specific capacity of Ni_3_S_2_–Ni@NC‐AE increased from 107.3 to 434.4 mAh g^−1^ after 150 cycles (for 45 min, 271 mAh g^−1^ after 150 cycles). Further increasing the etching time to 90 min, the specific capacity decreased to 181.8 mAh g^−1^. The above results illustrated that when etching time was less than 60 min, with the increase of etching time, Ni^0^ phase was etched, Ni_3_S_2_ active phase content gradually increased, as well as the amount of inter‐core void increased, which provided a large number of reaction active sites and potassium reservoir space, so the specific capacity increased. When etching time is too long, the specific capacity decreased due to the reduction of the active material content causing by excessive etching. Finally, 1.5 mg mL^−1^ of dopamine and 60 min etching time were chosen as the optimal conditions to prepare Ni_3_S_2_–Ni@NC‐AE anode. As shown in Figure 3g, the Ni_3_S_2_–Ni@NC‐AE had an excellent long‐term cycling stability at 1 A g^−1^ and maintained a specific capacity of 176.4 mAh g^−1^ after 5000 cycles. The potassium storage performance of Ni_3_S_2_–Ni@NC‐AE was better than that of many other transition metal chalcogenides‐based anodes previously reported, such as NiS@C,^[^ ^10^ ^]^ NiS_2_/3DGO,^[^ ^48^ ^]^ Ni‐Fe‐S CNT,^[^ ^49^ ^]^ and CuS‐C@Nb_2_O_5_‐C^[^ ^50^ ^]^ (Table S1, Supporting Information).

**Figure 4:**
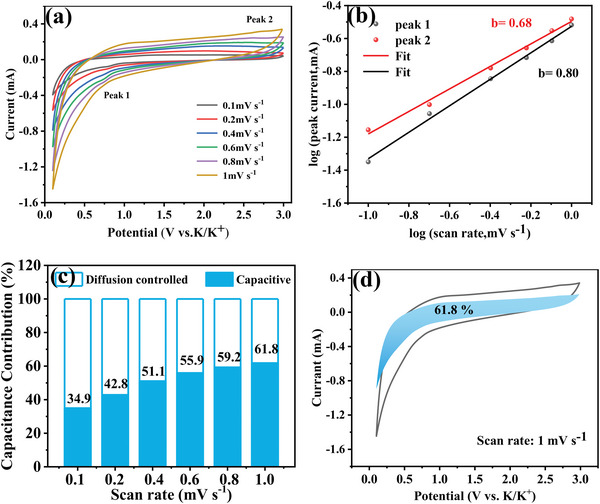
a) CV curves at various scan rates from 0.1 to 1 mV s^−1^. b) Determine the *b*‐value by fitting straight lines to the relationship between peak current and scan rate. c) Controlling ratio of capacitive and diffusive processes at different scan rates. d) Contribution ratio of capacitive processes at 1 mV s^−1^ (blue area)

To deeply probe the kinetic behavior of Ni_3_S_2_‐Ni@NC‐AE in PIBs, cyclic voltammetric (CV) tests were carried out in a voltage range of 0.1–3 V at different scan rates from 0.1 to 1 mV s^−1^ (**Figure** **4**a). The electrochemical energy storage process consisted of a diffusion process and a surface‐related pseudocapacitance process, which could be characterized by the relationship between the peak current (*i*) and the scan rate (*ν*) as the following Equation (1):^[^ ^10^, ^51^ ^]^

(1) $$i=a\nu^{b}$$

where *a* and *b* are constants in the formula. The *b*‐values for Peak 1 and Peak 2 were calculated as 0.80 and 0.68 (Figure 4b), respectively, which were between 0.5 and 1, implying a mixed contribution from both features.^[^ ^52^ ^]^ To further identify the specific contribution of the two behaviors, the current was fitted by diffusion process control current (*k*_2_ *v* ^1/2^) and surface‐related pseudocapacitance process control current (*k*_1_ *v*) with Equation (2):^[^ ^10^, ^52^ ^]^

(2) $$i\left(v\right)=k_{1}v+k_{2}v^{1/2}$$

The plot *i(v)/v* ^1/2^ against *v* ^1/2^ gives the values of *k*_1_ and *k*_2_, according which, *k*_2_ *v* ^1/2^ and *k*_1_ *v* could be obtained. As Figure 4c,d demonstrates, the pseudocapacitance contribution capacity increased with the increasing of scan speed, and it was 34.9% and 61.8% at 0.1 and 1 mV s^−1^, respectively. The results indicating that the pseudocapacitance contribution capacity dominated at high rate, which made the Ni_3_S_2_‐Ni@NC‐AE a good rate capability.^[^ ^52^ ^]^ The high pseudocapacitive contribution should be attributed to the excellent electrochemical kinetics of the nanospheres with intra‐core void structure (Figure 1) for potassium storage, which could provide more potassium storage reaction sites and shorten the K^+^ diffusion path.^[^ ^53^, ^54^, ^55^ ^]^

**Figure 5:**
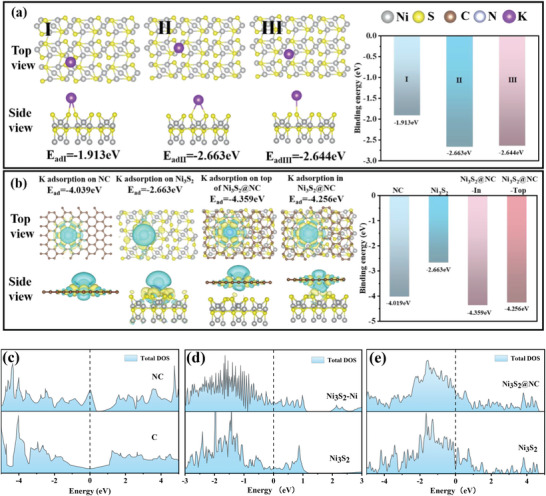
a) Side and top views of K atom adsorbed on different positions of Ni_3_S_2_. b) Charge difference density of models that K atoms adsorbed on NC, Ni_3_S_2_, inside Ni_3_S_2_@NC, and above Ni_3_S_2_@NC. Density of states (DOS) about NC and C (c); Ni_3_S_2_ and Ni_3_S_2_‐Ni (d); and Ni_3_S_2_ and Ni_3_S_2_@NC (e), respectively.

To gain a deep understanding of the effects of metallic Ni phase hybridization and N‐doped carbon encapsulation on K storage, DFT calculations were implemented to probe the model of K adsorption on Ni_3_S_2_, N‐doped carbon, and Ni_3_S_2_@NC, as well as the electronic properties. There were three possible sites for K adsorption on Ni_3_S_2_: the bridge site between S‐S (I), the top site for Ni atoms (II), and the top site for S atoms (III) (**Figure** **5**a). Calculations revealed that the adsorption energy of the II site (−2.663 eV) was higher than I site (−1.913 eV) and III site (−2.644 eV), implying that K adsorbed more easily to II site. Therefore, the adsorption site of the subsequent model was set at II. As shown in Figure 5b, the K adsorption energy was −2.663 and −4.039 eV on the separated Ni_3_S_2_ and NC layer model, respectively. The complex heterogeneous structure adsorption model manifested that both in the top adsorption (−4.256 eV) and interlayer adsorption (−4.359 eV), the adsorption energies were larger than those of the separation model, indicating that the composite structure of Ni_3_S_2_@NC captured K more efficiently compared to the single Ni_3_S_2_ and NC, leading to a stronger K storage capacity. The enhanced ability to anchor K^+^ was attributed to the synergistic effect between Ni_3_S_2_ and N‐doped carbon, additionally, the model of K^+^ adsorption between Ni_3_S_2_@NC layers displayed more pronounced electron annihilation (yellow) and electron aggregation (green), which also implied a more significant interaction effect on K^+^. The effects of metallic Ni phase hybridization, N‐doping, and N‐doped carbon shells on the electronic conduction were also effectively investigated by calculating the electronic density of states (DOS) of different systems. Figure 5c–e shows the DOS for the carbon and N‐doped carbon systems, Ni_3_S_2,_ and Ni_3_S_2_‐Ni hybrid systems, as well as Ni_3_S_2_ and Ni_3_S_2_@NC systems, respectively. Due to the introduction of the N element, the DOS of carbon located at the Fermi energy level was significantly increased and metallicity was enhanced. Additionally, the DOS around the Fermi energy level was increased overall after Ni_3_S_2_–Ni hybridization, demonstrating that metallic Ni phase effectively elevated the Ni_3_S_2_ electrical conductivity. Finally, although as DOS result showed, Ni_3_S_2_ had semiconductor‐characteristic, Ni_3_S_2_@NC heterostructure showed good electrical conductivity owing to the introduction of N‐doped carbon, enabling the material to be a good K‐ion storage anode.

## 3 Conclusion

N‐doped carbon encapsulated Ni_3_S_2_–Ni hybrid NPs with intra‐core void (Ni_3_S_2_–Ni@NC‐AE) were prepared based on simple electrodeposition of NiS NPs by regulating the electrocrystallization nucleation exclusion zone, dopamine coating, oxygen‐free annealing, and etching. The conductivity of the material is improved by metallic Ni hybridization in Ni_3_S_2_ and N‐doping in the carbon layer. Simultaneously, K^+^ mass transfer is accelerated due to the shortened transport path of the nanospheres and the intra‐core void structure, as well as the effect of the build‐in electric field of the core–shell structure. Additionally, K storage reaction kinetics are enhanced due to the increased K adsorption energy by the core‐shell heterostructure, abundant multi‐phase interface of Ni–Ni_3_S_2_, and large reaction surface area of nanopheres and inner‐core void structure. The volume expansion effectively was reduced by the sufficient buffer space and strong Ni–N chemical bonds provided by intra‐core void and yolk–shell structure. Consequently, the Ni_3_S_2_‐Ni@NC‐AE with fast electron/K^+^ transfer capability, superior reaction kinetics, and strong structural stability exhibits enhanced K‐ion batteries anode performance. The facile method based on electrodeposition provides a strategy to efficiently fabricate high‐performance metal nanosphere particles‐based multi‐structure energy storage materials.

## Conflict of Interest

The authors declare no conflict of interest.

## Supporting information

Supporting Information

## Data Availability

The data that support the findings of this study are available from the corresponding author upon reasonable request.
