# Supplementary material for: Ni3S2–Ni Hybrid Nanospheres with Intra‐Core Void Structure Encapsulated in N‐Doped Carbon Shells for Efficient and Stable K‐ion Storage
Source: Adv Sci (Weinh). 2023 Jan 1;10(5):2205556. doi: 10.1002/advs.202205556 (PMC9929274; doi:10.1002/advs.202205556)
Supplement: Supplementary file 1 — Supporting Information [file ADVS-10-2205556-s001.pdf]

ADVANCED SCIENCE
Open Access

Supporting Information

for *Adv. Sci.*, DOI 10.1002/advs.202205556

Ni_3S_2–Ni Hybrid Nanospheres with Intra-Core Void Structure Encapsulated in N-Doped Carbon Shells for Efficient and Stable K-ion Storage

Xiangtao Yu, Xiangyu Ren, Zhangfu Yuan, Xinmei Hou*, Tao Yang* and Mingyong Wang*

Electronic Supplementary Information

Ni_3S_2-Ni hybrid nanospheres with intra-core void structure encapsulated in N-doped carbon shells for efficient and stable K-ion storage

Xiangtao Yu[a,*], Xiangyu Ren[a], Zhangfu Yuan[a], Xinmei Hou[a,*], Tao Yang[a,*], Mingyong Wang[b]

[a]*Collaborative Innovation Center of Steel Technology, University of Science and Technology Beijing, Beijing 100083, P. R. China*

[b]*State Key Laboratory of Advanced Metallurgy, University of Science and Technology Beijing, Beijing 100083, P. R. China*

Experimental section

Electrodeposition of NiS nanosphere particles (NPs)

The loose porous NiS film composed of NiS NPs was electrodeposited at 25°C in a solution containing 0.2 M $NiSO_4·6H_2O$ (Aladdin, 98.5%), 0.2 M $Na_3C_6H_5O_7·2H_2O$ (Aladdin, 99%), 1 M $(NH_4)_2SO_4$ (Aladdin, 99%), 0.25 M $Na_2S_2O_3·5H_2O$ (Aladdin, 99%) at -2.5 A cm^{-2} for 60 s, by using a DC power. Cu and Pt foil with exposed area of 15 × 15 mm and 20 × 20 mm were used as working and counter electrode, respectively. The Cu foil was polished with sandpaper and cleaned with deionized water before electrodeposition.

Synthesis of Ni_3S_2-Ni hybrid NPs with intra-core void encapsulated by N-doped carbon (Ni_3S_2-Ni@NC-AE)

First, 100 mg as-obtained NiS NPs were ultrasonically dispersed in 100 mL Tris-

---

*Corresponding author. E-mail: xtyu2018@ustb.edu.cn (X.T. Yu)

*Corresponding author. E-mail: houxinmeiustb@ustb.edu.cn (X.M. Hou)

*Corresponding author. E-mail: yangtaoustb@ustb.edu.cn (T. Yang)

Buffer (10 mmol, pH 8.5) for 30 mins, subsequently, 150 mg dopamine was added in the solution and magnetically stirred for 12 h, the NiS NPs coated with polydopamine (NiS@PDA) were obtained. The NiS@PDA precipitates were separated by centrifugation and washed three times with deionized water and ethanol, respectively, and then dried in a vacuum oven at 80 °C for 12 h. The electrodeposited NiS and NiS@PDA were oxygen-free annealed in a tube furnace under 5%/95% H_2/Ar atmosphere at 450°C for 2 h to achieve phase transition and polydopamine carbonization to obtain N-doped carbon coated Ni_3S_2-Ni NPs (Ni_3S_2-Ni@NC). Finally, 100 mg Ni_3S_2-Ni@NC NPs were dispersed in a solution containing 37 mL deionized water, 12 mL alcohol, and 1.25 mL 4 M HCl, and stirred magnetically for 1 h to obtain N-doped carbon-encapsulated Ni_3S_2-Ni NPs with intra-core voids (Ni_3S_2-Ni@NC-AE). After centrifugation, Ni_3S_2-Ni@NC-AE was rinsed three times with deionized water and alcohol, and then dried in a vacuum oven at 80 °C for 12 h.

Structrucal characterization

Morphology and crystal structure of NiS-based NPs were studied by field-emission SEM (Zeiss Gemini500), TEM (JEM-2100F), HRTEM and X-ray diffraction (XRD, Rigaku D/max-RB X-ray Diffractometer) equipped with Cu Ka radiation (λ = 1.54059 Å) at 40 kV, respectively. The outer carbon shell information and mass ratio were recorded by Raman spectra (Renishaw 2000 Confocal Raman Microprobe) and TGA analysis, respectively. TGA was performed at a temperature range of 15°C-800°C with a heating rate of 10°C min^{-1}. The specific surface area and pore structure were examined with ASAP 2460 device by the Brunauer-Emmett-Teller (BET) method. X-ray

photoelectron spectroscopy (XPS) was performed on an ESCALAB 250Xi X-ray Photoelectron Spectrometer.

Electrochemical measurements

The working electrode was made by homogeneously mixing active materials (80 wt%), Super P (10wt%), and PVDF (10wt%) in the solvent N-methyl-2-pyrrolidinone (NMP) and then applying a slurry on carbon paper with an average loading mass of about 1.5 mg cm^{-2}. The carbon paper was immersed in 1 M HNO_3 for 12 h, then ultrasonically cleaned with deionized water for 20 min, and dried at 80°C for 24 h before use. The obtained electrodes were placed in vacuum oven dried at 80 °C for 12 h. Electrochemical characterization was performed using CR2032 coin cell, assembled in a glove box filled with argon gas (water and oxygen < 0.01 ppm). Coin cells were assembled using metal K as the counter electrode, glass fiber membrane (Whatman GF/D) as the separator, and 0.8 M KPF_6 dissolved in ethylene carbonate (EC) and diethyl carbonate (DEC) solution (EC : DEC = 1:1 Vol%) as the electrolyte. The electrochemical impedance spectroscopy (EIS) and cyclic voltammetric (CV) of 0.1-3 V vs K^+/K at different scan rates (0.2 - 1 mv s^{-1}) were recorded on a CHI 760E electrochemical workstation. The galvanostatic charge-discharge was carried out by using a NEWARE battery test system at 25 °C.

Density functional theory (DFT) calculations

All the DFT calculations were performed with Vienna *ab initio* simulation package (VASP) [S1]. The exchange-correlation potentials were obtained by the Perdew–Burke–Ernzerh (PBE) function of the generalized gradient approximation (GGA) [S2].

Implementation of the projected augmented wave (PAW) pseudopotential, and Van der Waals interactions are considered by the DFT-D3 approach [S3]. Monkhorst and Pack *k*-point meshes were used to complete Brillouin-zone integrations [S4]. The *k*-point of 1 × 2 × 1 was used for surface slabs and adsorption with different species. To simulate the effect of N-doped carbon on the properties Ni_3S_2, a hybrid slab structure was established with the surface of Ni_3S_2 (001) and a layer carbon, a vacuum layer of 15 Å to prevent undesired interactions. The cutoff energy was set to 450 eV, with an SCF energy tolerance for 10^{-5} eV per atom, when the combined forces on the atoms were less than -0.05 eV/Å, the calculation reached convergence. The adsorption energy of K atoms on the model was calculated as follows:

$$\Delta E_a = E_{(slab+k)} - E_{slab} - E_k$$

where $E_{(slab+k)}$ was the overall energy of the K atom adsorption on the slab model. $E_{(slab)}$ is the total energy of the slab model and E_k is the energy of a single K atom. the Slab model included Ni_3S_2 (001), NC (001), C (001) and Ni_3S_2@NC.

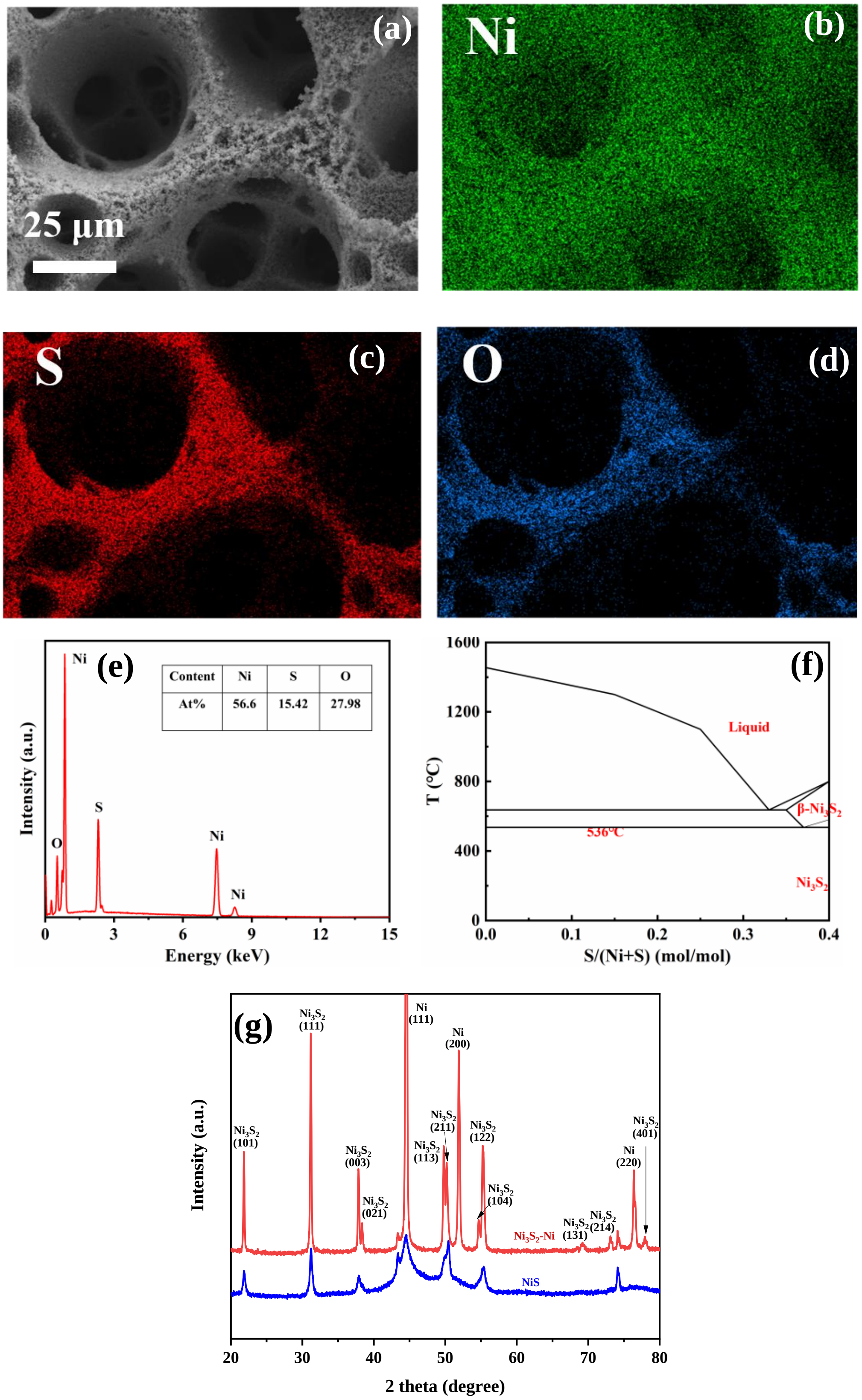

Fig. S1. SEM (a), corresponding EDS mapping images (b, c, d), and spectra (e) of electrodeposited NiS. (f) is Ni-S binary phase diagram. (g) is XRD diffractogram of

electrodeposited NiS before (NiS) and after (Ni_3S_2) annealing.

EDS results showed that the deposited samples were mainly composed of Ni and S elements with a mole ratio (Ni:S) of about 4:1; in addition a small amount of O was observed (fig. S1), which may be due to oxidation of the film surface [S5]. It could found that Ni_3S_2-Ni could be obtained after annealing the electrodeposited NiS at 450 °C under Ar/H_2 atmosphere.

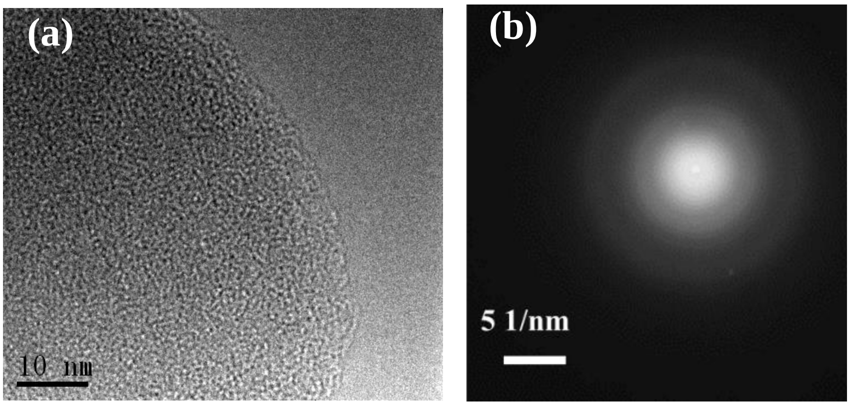

Fig. S2. HRTEM (a) and corresponding SAED (b) images of electrodeposited NiS.

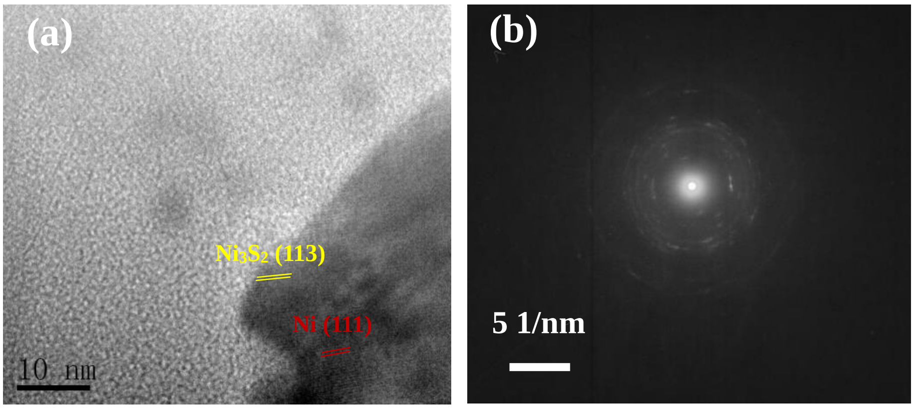

Fig. S3. HRTEM (a) and corresponding SAED (b) images of Ni_3S_2-Ni@NC self-polymerization in Tris-Buffer with dopamine concentrations of 1.5 mg ml^{-1}.

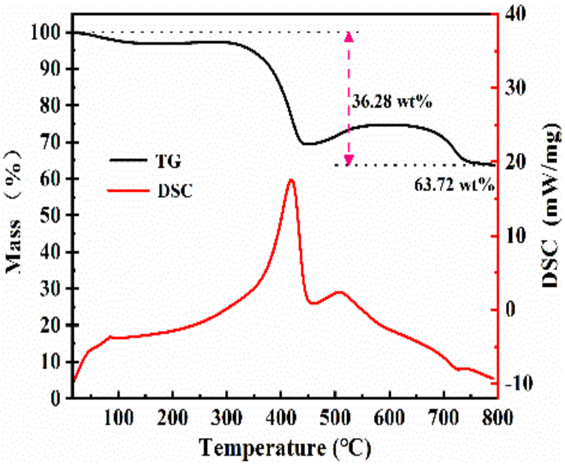

Fig. S4. TG and DSC plots of Ni_3S_2-Ni@NC-AE.

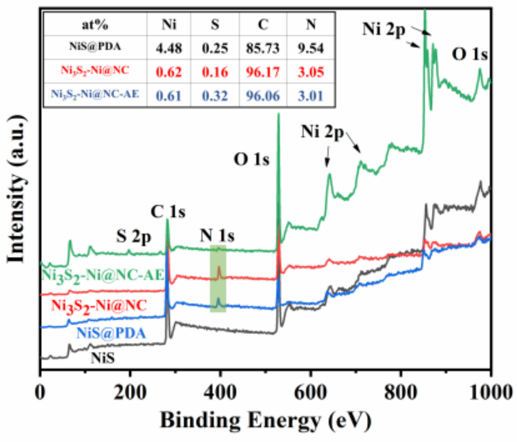

Fig. S5. Survey XPS spectra of NiS, NiS@PDA, Ni_3S_2-Ni@NC, and Ni_3S_2-Ni@NC-AE.

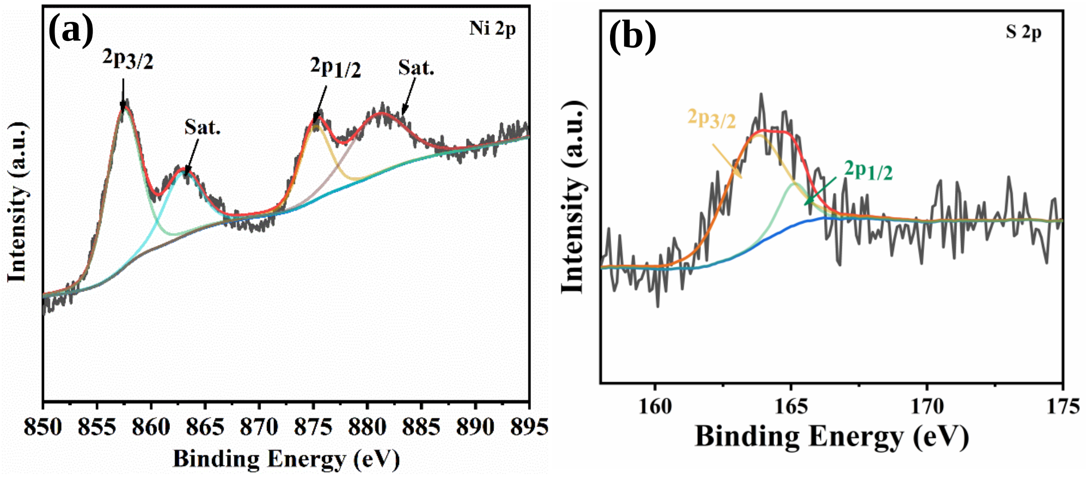

Fig. S6. High-resolution XPS spectra of Ni (a)elements and S (b)elements of electrodeposition NiS

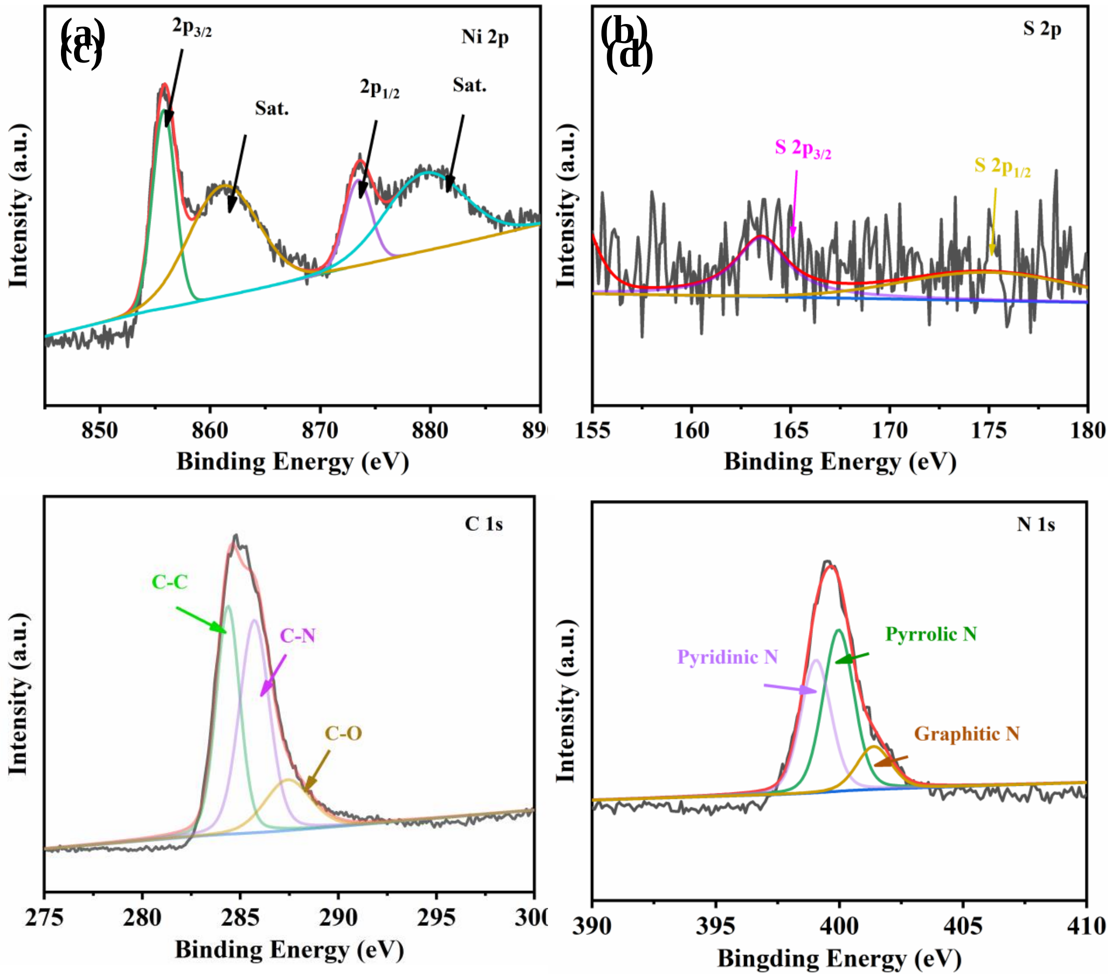

Fig. S7. High-resolution XPS spectra of Ni (a) S (b) C (c) N (d) elements of NiS@PDA

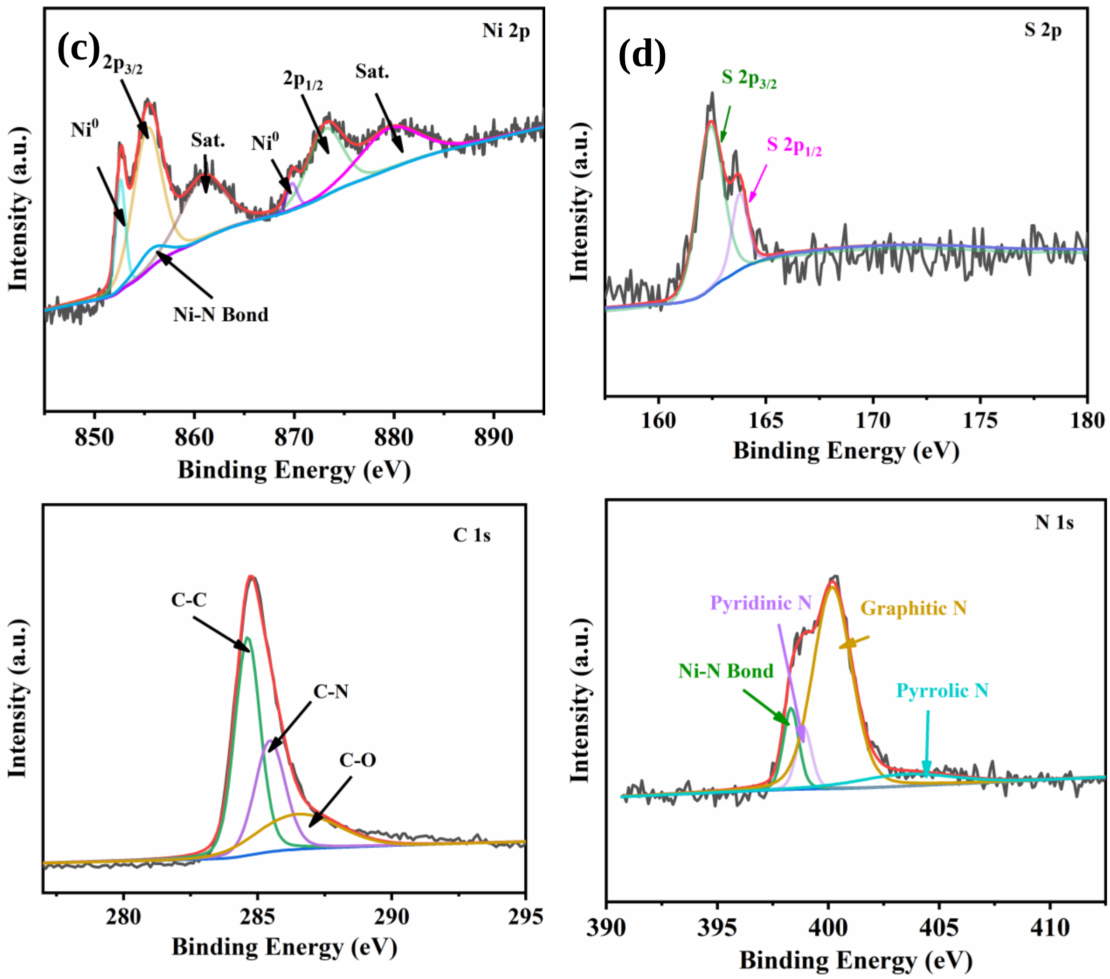

Fig. S8. High-resolution XPS spectra of Ni (a) S (b) C (c) N (d) elements of Ni_3S_2-Ni@NC

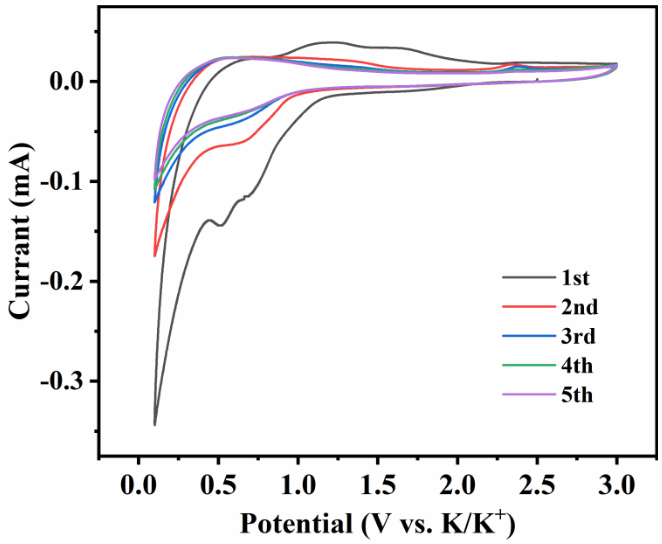

Fig. S9. CV of electrodeposited NiS in the voltage window between 0.1-3V with a sweep rate of 0.2mV S^{-1}

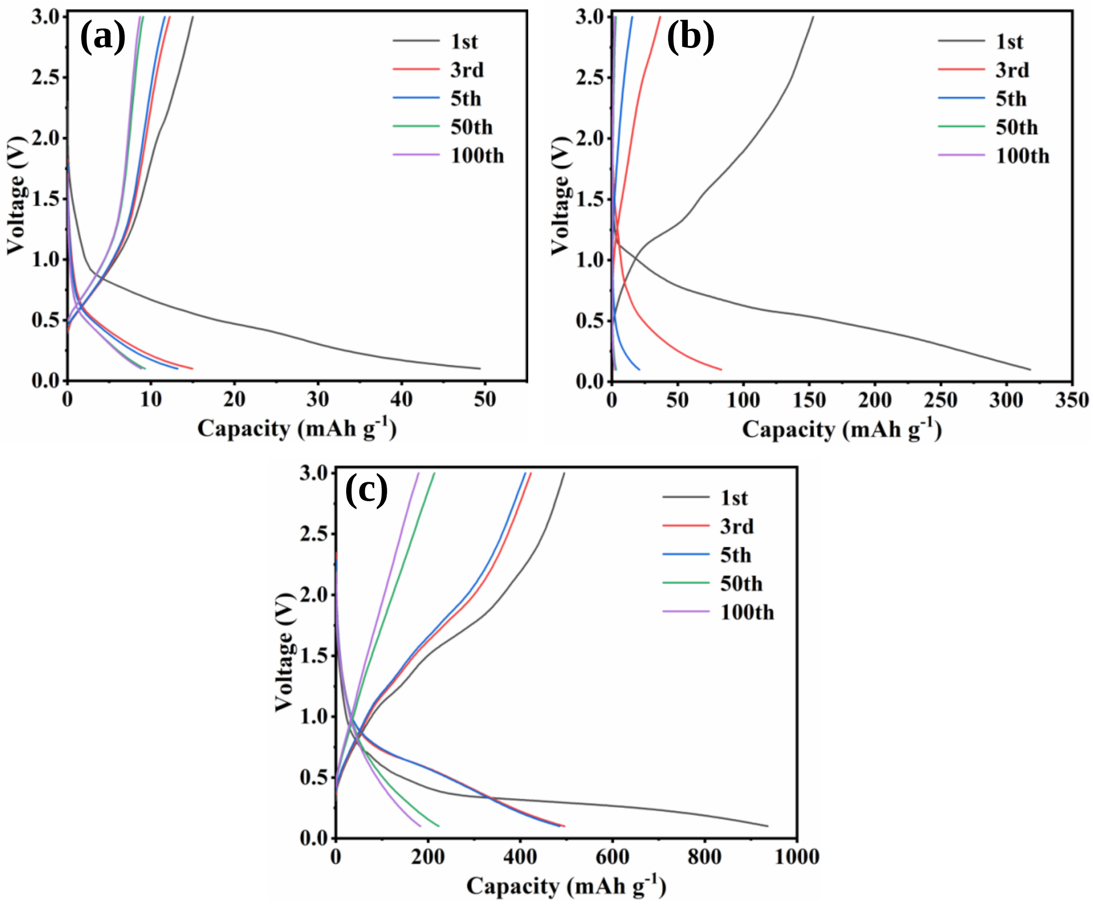

Fig. S10. Charge and discharge curves of electrodeposited NiS (a) NiS@PDA (b) Ni_3S_2-Ni@NC (c).

NiS and NiS@PDA exhibited very low capacities after 50 cycles (NiS: 3.2 mAh g^{-1}, NiS@PDA: 19.2 mAh g^{-1}), while Ni_3S_2-Ni@NC maintained a higher capacity of 223 mAh g^{-1} after 50 cycles, however, the capacity dropped significantly to 183.1 mAh g^{-1} after 100 cycles. This suggested the intranuclear void structure produced by etching could sufficiently buffer the volume change to improve cycling stability.

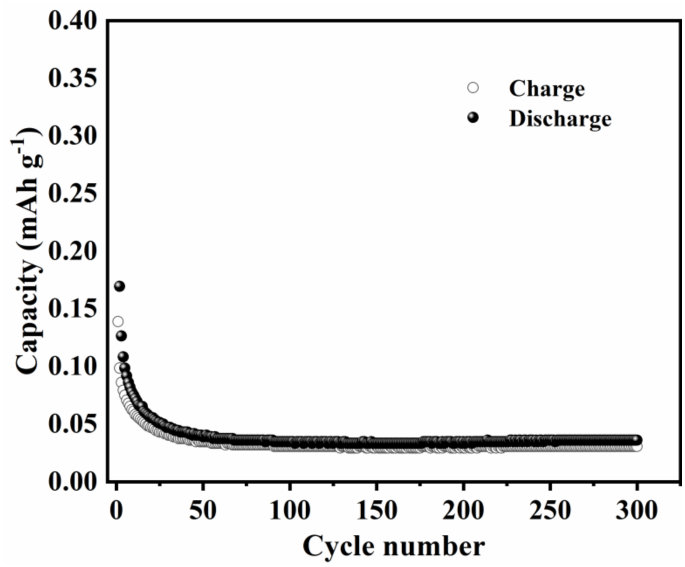

Fig. S11. Cycle curve of carbon paper substrate at 0.1A g^{-1}

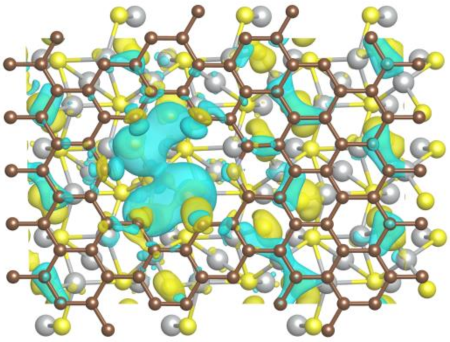

Fig. S12. Charge difference density of Ni_3S_2@NC heterostructure (top view).

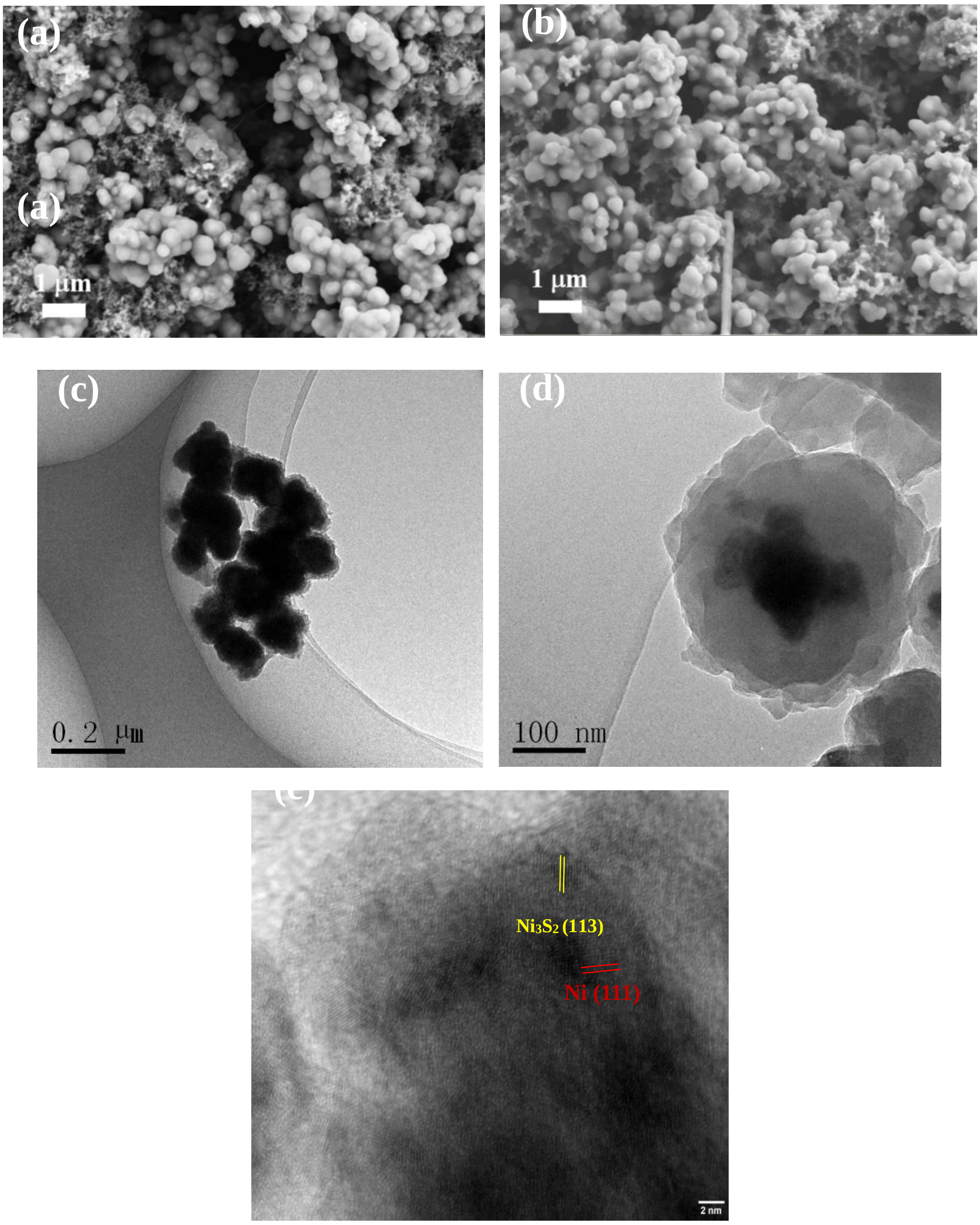

Fig. S13. SEM images of Ni_3S_2-Ni@NC-AE electrode (a) before and (b) after 500 cycles at 0.1 A g^{-1}. (c), (d) and (e) are the TEM images with different magnification and HRTEM image of Ni_3S_2-Ni@NC-AE after 500 cycles at 0.1 A g^{-1}, respectively.

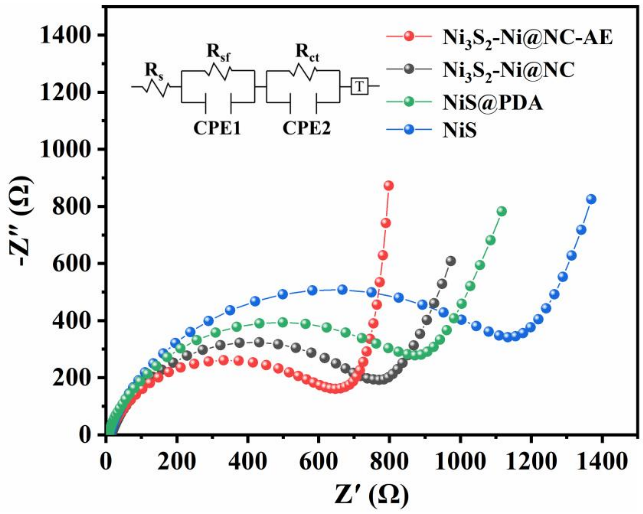

Fig. S14. EIS plots of different samples and the corresponding fitted circuit diagrams.

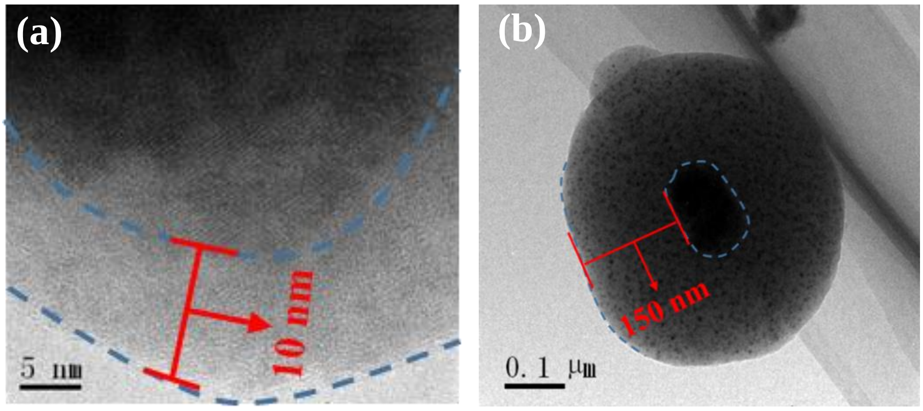

Fig. S15. HRTEM and TEM of Ni_3S_2-Ni@NC-AE with dopamine concentrations of (a) 1mg ml^{-1} and (b) 2 mg ml^{-1}.

Table S1. Comparison of potassium storage performance between this work and other similar anode materials.

Electrode materials	Current density ($A\ g^{-1}$)	Reversible capacity ($mA\ h\ g^{-1}$)	Cycle number	References
**Ni_3S_2-Ni@NC-AE**	**0.1**	**434.4**	**150**	**This work**
	**1**	**180.5**	**500**	
**NiS@C**	**0.1**	**456**	**50**	[S6]
	**1**	**171**	**300**	
**$NiCo_2S_4$@N-HCNFs**	**0.1**	**263.7**	**200**	[S7]
	**3.2**	**134.3**	**600**	
**$Ni_2P_{0.75}S_{0.25}$/Ni@C/G**	**0.15**	**372**	**200**	[S8]
**NiS_2/3DGO**	**0.1**	**398**	**50**	[S9]
	**1**	**260**	**160**	
**ACF/NiS**	**0.5**	**292.5**	**100**	[S10]
	**0.5**	**122**	**300**	
**Flower NiS_2**	**0.2**	**433**	**100**	[S11]
	**1**	**239**	**500**	
**Ni_2P@NPC**	**0.1**	**282**	**100**	[S12]
	**1**	**212**	**5000**	
**NiS_2@C@C**	**0.05**	**302.7**	**100**	[S13]
	**0.5**	**116.9**	**200**	
**Ni-Fe-S CNT**	**0.1**	**181**	**50**	[S14]
**Y-S NiSx@C**	**0.1**	**415**	**50**	[S15]
**NiS2-C@Nb2O5-C**	**0.1**	**369.1**	**100**	[S16]

The mass ratio of Ni_3S_2,Ni and C were calculated as follows:

Firstly, We assumed that Ni, Ni_3S_2 and C are x, y and z mg, respectively. Total mass is 1 mg.

So the equations are as follow:

$$\mathrm{x + y + z = 1}$$

$$\frac{x}{x+y} = 0.4$$

$$\frac{x}{58.7} \times 74.7 + \frac{y}{240} \times 3 \times 74.7 = 0.6372$$

Reference

[S1] G. Kresse, J. Furthmuller, Efficiency of ab-initio total energy calculations for metals and semiconductors using a plane-wave basis set. Comput. Mater. Sci., 1996, 6, 15–50.

[S2] J. P. Perdew, K. Burke, M. Ernzerhof, Perdew, burke, and ernzerhof reply. Phys. Rev. Lett., 1998, 80, 891.

[S3] S. Grimme, J. Antony, S. Ehrlich, H. Krieg, A consistent and accurate ab initio parametrization of density functional dispersion correction (DFT-D) for the 94 elements H-Pu. J. Chem. Phys., 2010, 132, 154104.

[S4] H. J. Monkhorst, J. D. Pack, Special points for Brillouin-zone integrations. Phys. Rev. B: Solid State, 1976, 13, 5188–5192.

[S5] X. Huang, Z. Zhao, L. Cao, Y. Chen, E. Zhu, Z. Lin, M. Li，A. Yan, A. Zettl, Y. Morris Wang, X. Duan, T. Mueller, Y. Huang, High-performance transition metal–doped Pt3Ni octahedra for oxygen reduction reaction. Science, 2015, 348(6240) 1230-1234.

[S6] Xu Zhao, Feiyan Gong, Yundong Zhao, Bin Huang, Dong Qian, Hong-En Wang, Wenhua Zhang, Zhijian Yang, Encapsulating NiS nanocrystal into nitrogen-doped carbon framework for high performance sodium/potassium-ion storage. Chemical Engineering Journal 392 (2020) 123675.

[S7] W. Zhang, J. Chen, Y. Liu, S. Liu, X. Li, K. Yang, L. Li, Decoration of hollow nitrogen-doped carbon nanofibers with tapered rod-shaped NiCo2S4 as a 3D structural

high-rate and long-lifespan self-supported anode material for potassium-ion batteries. J. Alloy Compd. 823, 2020, 153631.

[S8] X. Zhao, Y. Song, Z. Liu, Kinetics enhanced hierarchical Ni2P1−xSx/Ni@ carbon/graphene yolk–shell microspheres boosting advanced sodium/potassium storage. J. Mater. Chem. A, 2020, 8, 23994-24004.

[S9] K. Han, J. Meng, X. Hong, X. Wang, L. Mai, Three-dimensional graphene-supported nickel disulfide nanoparticles promise stable and fast potassium storage. Nanoscale, 2020, 12, 8255-8261.

[S10] W. Wei, F. Wang, J. Yang, J. Zou, J. Li, K. Shi, A Superior Potassium-Ion Anode Material from Pitch-based Activated Carbon Fibers with Hierarchical Pore Structure Prepared by Metal Catalytic Activation. ACS Appl. Mater. Interfaces, 2021, 13(5) 6557-6565.

[S11] S. Liang, H. Shi, Z. Yu, Q. Liu, K. Cai, J. Wang, Z. Xu, Uncovering the design principle of conversion-based anode for potassium ion batteries via dimension engineering. Energy Storage Mater., 34, 2021, 536-544.

[S12] Z. Yan, Z. Huang, Y. Yao, X. Yang, H. Li, C. Xu, Y. Kuang, H. Zhou, Monodispersed Ni2P nanodots embedded in N, P co-doped porous carbon as super stable anode material for potassium-ion batteries. J. Alloy Compd., 858, 2021, 158203.

[S13] L. Yang, W. Hong, Y. Zhang, Y. Tian, X. Gao, Y. Zhu, G. Zou, H. Hou, X. Ji, Hierarchical NiS2 Modified with Bifunctional Carbon for Enhanced Potassium-Ion Storage. Adv. Funct. Mater. 29(50), 2019, 1903454.

[S14] S. Zhang, G. Wang, B. Wang, J. Wang, J. Bai, H. Wang, 3D Carbon Nanotube Network Bridged Hetero-Structured Ni-Fe-S Nanocubes toward High-Performance Lithium, Sodium, and Potassium Storage. Adv. Funct. Mater., 30(24), 2020, 2001592.

[S15] Q. Yao, J. Zhang, J. Li, W. Huang, K. Hou, Y. Zhao, L. Guan, Yolk–shell NiS x@ C nanosheets as K-ion battery anodes with high rate capability and ultralong cycle life. J. Mater. Chem. A, 2019, 7, 18932-18939.

[S16] K. Cao, R. Zheng, S. Wang, J. Shu, X. Liu, H. Liu, K. Huang, Q. Jing, L. Jiao, Boosting Coulombic Efficiency of Conversion-Reaction Anodes for Potassium-Ion Batteries via Confinement Effect. Adv. Funct. Mater., 30(52), 2020, 2007712.
